# A Role of Ginseng and Its Constituents in the Treatment of Central Nervous System Disorders

**DOI:** 10.1155/2016/2614742

**Published:** 2016-08-18

**Authors:** Natasya Trivena Rokot, Timothy Sean Kairupan, Kai-Chun Cheng, Joshua Runtuwene, Nova Hellen Kapantow, Marie Amitani, Akinori Morinaga, Haruka Amitani, Akihiro Asakawa, Akio Inui

**Affiliations:** ^1^Department of Psychosomatic Internal Medicine, Kagoshima University Graduate School of Medical and Dental Sciences, Kagoshima 890-8544, Japan; ^2^Faculty of Medicine, Sam Ratulangi University, Manado 95115, Indonesia

## Abstract

Ginseng, a perennial plant belonging to the* Panax* genus of the Araliaceae family, has been used in China, Korea, and Japan as a traditional herbal medicine for thousands of years. Ginseng is recorded to have exhibited a wide variety of beneficial pharmacological effects and has become a popular and worldwide known health supplement and drug. The protective effects of ginseng on central nervous system are discussed in this review. Ginseng species and ginsenosides and their intestinal metabolism and bioavailability are concisely introduced. The molecular mechanisms of the effects of ginseng on central nervous system, mainly focused on the neuroprotection properties of ginseng, memory, and learning enhanced properties, and the effects on neurodegenerative disorders are presented. Thus, ginseng and its constituents are of potential merits in the treatment of cerebral disorders.

## 1. Introduction

Ginseng has a medical history for thousands of years and become one of the most widely used traditional herbal medicines [1]. It belonged to the* Panax* genus of the Araliaceae family. The word* Panax* means “all heal” in Greek, which is based on the view that ginseng is powerful to heal any kind of disease. Ginseng is originated from the Chinese words “*Jen Sheng*,” meaning “man-herb,” because the shape in root of the plant resembles a humanoid form. The most extensively investigated ginsengs are* Panax ginseng* (Korean ginseng),* Panax quinquefolius *L. (American ginseng), and* Panax notoginseng* (Chinese ginseng) [2]. It has been documented that ginseng and its constituents exhibit a wide variety of beneficial pharmacological effects. Constituents of ginseng plant have been shown to produce adaptogenic, restorative, vasodilatory, immunomodulatory, anti-inflammatory, antioxidant, antiaging, anticancer, antifatigue, antidiabetic, antistress, and antidepressive effects in animals and humans [3–8].

Ginseng is also known to affect the nervous system, due to various effects that are beneficial to brain. Ginsenosides and other active constituents from ginseng are known to show neuroprotective properties and worked as cognitive performance and memory enhancer [9, 10]. The purpose of this review is to discuss the effects of ginseng on central nervous system, mainly focused on the neuroprotection properties of ginseng, memory, and learning enhanced properties, and the effects on neurodegenerative disorders.

## 2. Chemical Structure and Component

**Figure 1:**
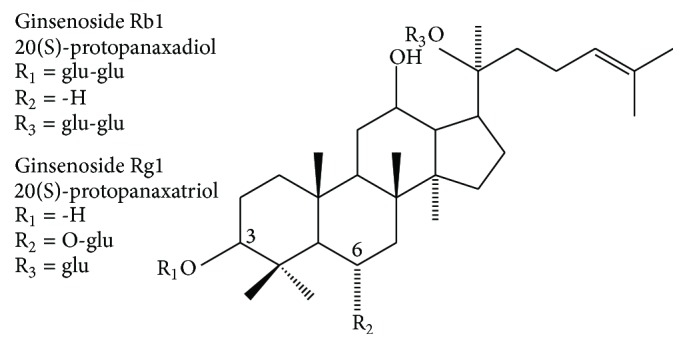
Structures of ginsenosides Rb1 and Rg1. Based on the chemical structure, there are two major structural classes: the protopanaxadiol (PPD) and protopanaxatriol (PPT). Ginsenoside Rb1 is an example of PPD type and ginsenoside Rg1 is an example of PPT type.

The major active compounds in ginseng are triterpenoid glycosides, known also as the ginsenosides, contained in the roots, leaves, stems, flower buds, and berries. Ginsenosides are considered part of the defense mechanism in ginseng plants [11–16]. Identification and quantification of ginsenoside from ginseng plants have been established [17]. Ginsenosides consist of a 4-ring steroid backbone structure [18, 19]. To date, more than 100 types of ginsenosides have been identified and isolated from the various parts of ginseng [9, 20–23]. Sugar types, quantities, and attachment positions, changeable carbon (C)-20 side chain, and stereoisomerism are the differentiating factors between each of ginsenosides [19, 24]. Generally, there are two major groups of ginsenosides: protopanaxadiols (PPD), including Rb1, Rb2, Rc, Rd, Rg3, Rh2, and Rh3; protopanaxatriols (PPT), including Re, Rf, Rg1, Rg2, and Rh1; and there is also the nonsteroidal saponin, oleanic acid group, which contained one ginsenoside, Ro [25]. Difference between two groups is the attached position of sugar moieties. In PPD group, the sugar moieties are attached to the B-OH at C-3 and/or C-20, while in the PPT group they are attached to a-OH at C-6 and/or C-20 (Figure 1) [26, 27].

Besides the ginsenosides, other components are also found in ginseng, such as polysaccharides, flavonoids, volatile oils, and the recently identified nonsaponin compound called gintonin [24, 28].

## 3. Bioavailability

The oral bioavailability of ginsenosides is very poor. It cannot be easily absorbed by the intestines due to their hydrophilicity [29]. The absorption of ginsenosides in the intestinal mucosa is energy-dependent [30–32], and its availability of both intact ginsenosides and/or its metabolites from the intestines are very low [33–35]. Biotransformation of ginsenosides by microbiota in gut may form the deglycosylated products [36]. The deglycosylated products are more permeable and absorbable than ginsenosides [37]. However, the extensive biliary excretion through active transport causes the shortage of its biological half-life to result in a low systemic exposure level [36].

Some studies has been developed to overcome this problem, such as coadministration with adrenalin [38] or using lipid-based formulations [39, 40] and the suppression of p-glycoprotein efflux system [30] that are proven to increase the oral bioavailability of ginsenosides.

## 4. Effects on the Central Nervous System

**Figure 2:**
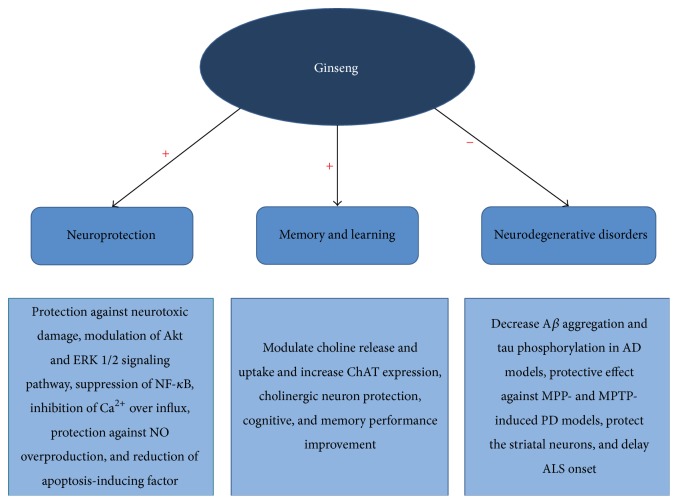
Multiple therapeutic targets of ginseng and its metabolites in central nervous system. ERK 1/2: extracellular-signal-regulated kinases 1 and 2, NF-*κ*B: nuclear factor-kappa B, NO: nitric oxide, ChAT: choline acetyltransferase, A*β*: *β*-amyloid, AD: Alzheimer's disease, MPTP: 1-methyl-4-phenyl-1,2,3,6-tetrahydropyridine, MPP: 1-methyl-4-phenylpyridium, PD: Parkinson's disease, and ALS: amyotrophic lateral sclerosis.

Ginseng and its constituents are known to have the beneficial effects on central nervous system (CNS) disorders including the cognitive performance, memory, and neurodegenerative diseases (Figure 2).

### 4.1. Neuroprotection

Many studies have identified the neuroprotective properties of ginseng and ginsenosides [41]. Ginsenosides Rb1 and Rg1 play a major role in neuroprotective effect. Rb1 was shown to increase the neuron cell survival and improve neurite growth [42]. Rb1 protects hippocampal neuron from the ischemic damage and also delays the neuronal death from transient forebrain ischemia [19]. Rg1 exerts a protective effect against the transient focal cerebral ischemic injury in rats with cerebral injury [43] and also protects AB25-35-induced cortical neuron apoptosis through the downregulation of nuclear factor-kappa B (NF-*κ*B)/nitric oxide (NO) signaling pathway [44]. An increase of membranes fluidity was observed in both Rb1 and Rg1 experiments [19], Rb1 enhanced the membrane fluidity of cortical cells in rats [45], and Rg1 increased the fluidity of synaptosomal membranes impaired by FeSO4-cysteine [46].

Otherwise, ginsenosides Rd and Re also have neuroprotective properties. Ginsenoside Rd is shown to ameliorate ischemic stroke-induced damage and prolong the neural cells' survival through several mechanism [47], such as phosphoinositide-3-kinase/AKT and phosphoextracellular signal-regulated protein kinase (ERK) 1/2 pathways [48], suppression of the NF-*κ*B, transient receptor potential melastatin, acid sensing ion channels 1a [49], poly(ADP-ribose) polymerase-1 [50], protein tyrosine kinase activation, the upregulation of the endogenous antioxidant system, preservation of mitochondrial membrane potential, and reduction of cytochrome c-releasing and apoptosis-inducing factor [51–53]. Recent study suggested that Rd also promotes the neurites outgrowth, an important process for neuronal repair, of PC12 cells through upregulating GAP-43 expression via ERK- and ARK-dependent signaling [54]. Ginsenoside Re has been reported to decrease the mitochondrial swelling and prevent the reduction of H(+)-ATPase activity in cerebral ischemia-reperfusion injury in rats [55].

### 4.2. Memory and Learning

Ginseng and its constituents have significant effects on memory and cognitive performances. Local administration of ginseng in brain-damaged rats showed a significant improvement in learning and memory [25]. Ginsenoside Rb1 has been reported to increase the uptake of choline in cerebral cholinergic nerve endings [56] and modulate acetylcholine (Ach) release and uptake [57], which related to learning process and memory. Rb1 is also beneficial to cognitive impairment and hippocampus senescence [58]. Both Rb1 and Rg1 have been shown to improve the scopolamine-induced amnesia in rodents and also elevate the level of choline acetyltransferase (ChAT) in rodent brains [59, 60]. Ginseng prevented the advance glycation end product- (AGE-) induced memory impairment by decreasing the expression of receptors for AGE (RAGEs) and *κ*-light-chainenhancer of activated B cells (NF-*κ*B) [61]. Chronic administration of ginsenoside to mice averts the memory lost and impairment [62, 63]. Rg3 improved learning and memory impairments in lipopolysaccharide-induced cognitive impairment [64]. Rg3 and Rg5/Rg1 administration were also shown to enhance memory in scopolamine or ethanol-induce memory dysfunction in mice [65].

Other compounds like gintonin also possessed the ability to improve cognitive functions. Systemic administration of gintonin showed an improvement in contextual memory formation at molecular level up to behavioral level in experimental mice [66].

### 4.3. Neurodegenerative Diseases

The merits of ginseng and ginsenosides also included the neurodegenerative diseases (Table 1). Neurodegenerative diseases are associated with progressive loss of structure or neuron function, with loss of cognitive function and motor disabilities. Neurodegenerative diseases include Alzheimer's disease (AD), Parkinson's disease (PD), Huntington's disease (HD), and amyotrophic lateral sclerosis (ALS).

#### 4.3.1. Alzheimer's Disease

AD accounts for more than 60–70% of dementia, a general term for a memory disorder, including the loss of memory, and other intellectual abilities, which are great enough to interfere with the person's daily life. One of the major pathological features of AD is the presence of *β*-amyloid (A*β*) around arterioles or capillaries wall in the brain [81]. Administration of ginseng and its constituents may inhibit A*β* aggregation in cultured neurons. Ginsenoside Rg1 administration showed a significant reduction in cerebral A*β* in aged transgenic AD mice, with improved spatial learning abilities and memory [67]. Gintonin also is shown to improve AD, by attenuating the deposition of amyloid plaque, and memory impairment in AD mouse [68]. A*β* peptides resulted from the amyloid precursor protein (APP), cleaved by beta secretase (BACE1) and gamma secretase. Several studies have showed that ginseng and ginsenosides have the abilities to enhance the nonamyloidogenic processing of APP by increasing *α* secretase activities and decrease the amyloidogenic processing by decreasing BACE1 [68, 69].

Hyperphosphorylated tau protein is also known to cause AD. Hyperphosphorylation of tau protein will lead to the accumulation of neurofibrillary tangles inside nerve cell bodies. This event will lead to an interference of the cellular transport process in brain [82]. Total ginsenosides extracted from stems and leaves of* Panax ginseng* are shown to inhibit tau hyperphosphorylation by enhancing the phosphatase activity of purified calcineurin in SY5Y cells [70]. Ginsenosides Rd and Rb1 are also shown to reduce hyperphosphorylated tau by enhancing phosphatase 2A level (PP2A) [71]. Rg1 was shown to reverse the memory impairments by decreasing hyperphosphorylated tau and suppressing A*β* formation in rat brain [83].

A decrease of cholinergic neurons in brain is associated with AD. Ginsenoside Rg5 has been shown to improve cognitive dysfunction and neuroinflammation and modulate both AChE and ChAT activity in brain cortex [72]. Other ginsenosides, Re and Rd, are also shown to enhance ChAT and vascular acetylcholine transporter (VAChT) to increase the Ach level in neuro-21 cells [73].

#### 4.3.2. Parkinson's Disease

PD is a neurodegenerative disorder affecting mainly the motor system, as a result of the death of dopaminergic neurons in substantia nigra (SN). Administration of 1-methyl-4-phenyl-1,2,3,6-tetrahydropyridine (MPTP) or its metabolite 1-methyl-4-phenylpyridium (MPP^+^) has been used to induce PD models in various animal studies. MPTP and MPP^+^ destroy dopaminergic neurons in substantia nigra, which causes PD symptoms. Treatment of ginseng extracts produced neuroprotective effect in PD mouse model [74, 84]. Rb1 and Rg1 are shown to inhibit the decrease of neurite length or numbers in MPP-treated primary dopaminergic cultures [75]. Rg1 has been reported to reduce MPTP-induced substantia nigra neuronal loss in C57/BL6 mice [74, 76]. Oral intake of ginseng extract, G115, significantly prevented tyrosine hydroxylase-positive cell loss in substantia nigra and attenuated the locomotor dysfunction in MPTP treated rodents [77]. G115 has also been shown to reduce dopaminergic cell loss in *β*-sitosterol *β*-d-glucoside fed rats (BSSG rat model of Parkinson's disease) [78].

#### 4.3.3. Other Neurodegenerative Diseases

In addition to the effects on AD and PD, ginseng and its constituent also showed similar influence to other neurodegenerative diseases, including Huntington's diseases and amyotrophic lateral sclerosis. Ginsenosides have been shown to protect the striatal neurons in cellular models of HD [79]. Moreover, ginseng extract also delayed the ALS onset in B6SJL-TgN(SOD1-G93A)1Gur transgenic mice [80]. But further studies are still required to determine the effectiveness and elucidate their mechanisms of action of ginseng or its constituents in other neurodegenerative diseases.

## 5. Conclusions and Future Perspective

Ginseng has been used for thousands of years as traditional medicine. The results reviewed above from cell culture systems, animal studies, and human studies suggest that ginseng and its constituents are effective to produce the beneficial effects on CNS, including neuroprotection, cognitive, and memory performance enhancement. However, the active compounds, ginsenosides and gintonin, which influenced the CNS have not been fully elucidated. Further studies are still necessary to unravel the mechanisms of action, detailed pharmacokinetics and toxicity, standardization of each ginseng preparation, and therapeutic doses in animals and humans. Additionally, the clinical trials are still required to confirm the effectiveness of ginseng and its constituents in modulating these neurodegenerative diseases. Overall, ginseng and its constituents are merits in the treatment of cerebral disorders.

## Figures and Tables

**Table 1:** Effects of ginseng and its metabolites in neurodegenerative disorder. AD: Alzheimer's disease, PD: Parkinson's disease, HD: Huntington's disease, ALS: amyotrophic lateral sclerosis, and A*β*: *β*-amyloid.

Neurodegenerative disorder	Active compound	Research type	Mechanism	Reference
AD	Rb1, Rg1, Rg5, Rd, Re, gintonin, and ginseng extract panaxynol	Animal model and cell culture	Decrease A*β* production and aggregation, increase A*β* clearance, decrease of tau hyperphosphorylation, and improve cholinergic function	[67–73]

PD	Rb, Rg1, and ginseng extract G115	Animal model and cell culture	Protection against neurotoxic damage and inhibition of a-synuclein aggregation	[74–78]

HD	Rb1, Rc, and Rg5	Animal model	Protection against neurotoxic damage and inhibition of Ca^2+^ signaling	[79]

ALS	*Panax quinquefolius*'s root extract	Animal model	Unknown	[80]
